# Effects of Selective Deletion of Tyrosine Hydroxylase from Kisspeptin Cells on Puberty and Reproduction in Male and Female Mice

**DOI:** 10.1523/ENEURO.0150-17.2017

**Published:** 2017-06-22

**Authors:** Shannon B. Z. Stephens, Melvin L. Rouse, Kristen P. Tolson, Reanna B. Liaw, Ruby A. Parra, Navi Chahal, Alexander S. Kauffman

**Affiliations:** Department of Reproductive Medicine, University of California, San Diego La Jolla, CA 92093

**Keywords:** dopamine, GnRH, Kiss1, kisspeptin, puberty, reproduction

## Abstract

The neuropeptide kisspeptin, encoded by *Kiss1*, regulates reproduction by stimulating GnRH secretion. *Kiss1-*syntheizing neurons reside primarily in the hypothalamic anteroventral periventricular (AVPV/PeN) and arcuate (ARC) nuclei. AVPV/PeN *Kiss1* neurons are sexually dimorphic, with females expressing more *Kiss1* than males, and participate in estradiol (E_2_)-induced positive feedback control of GnRH secretion. In mice, most AVPV/PeN *Kiss1* cells coexpress tyrosine hydroxylase (TH), the rate-limiting enzyme in catecholamine synthesis (in this case, dopamine). Dopamine treatment can inhibit GnRH neurons, but the function of dopamine signaling arising specifically from AVPV/PeN *Kiss1* cells is unknown. We generated a novel TH flox mouse and used Cre-Lox technology to selectively ablate *TH* specifically from *Kiss1* cells. We then examined the effects of selective *TH* knock-out on puberty and reproduction in both sexes. In control mice, 90% of AVPV/PeN *Kiss1* neurons coexpressed *TH*, whereas in mice lacking *TH* exclusively in *Kiss1* cells (termed Kiss THKOs), *TH* was successfully absent from virtually all *Kiss1* cells. Despite this absence of *TH*, both female and male Kiss THKOs displayed normal body weights, puberty onset, and basal gonadotropin levels in adulthood, although testosterone (T) was significantly elevated in adult male Kiss THKOs. The E_2_-induced LH surge was unaffected in Kiss THKO females, and neuronal activation status of kisspeptin and GnRH cells was also normal. Supporting this, fertility and fecundity were normal in Kiss THKOs of both sexes. Thus, despite high colocalization of *TH* and *Kiss1* in the AVPV/PeN, dopamine produced in these cells is not required for puberty or reproduction, and its function remains unknown.

## Significance Statement

Kisspeptin promotes reproduction, and kisspeptin neurons in the hypothalamic anteroventral periventricular (AVPV/PeN) nucleus mediate estradiol (E_2_)-positive feedback control of GnRH secretion. Dopamine treatment can inhibit GnRH neurons, and most AVPV/PeN kisspeptin cells are also dopaminergic (coexpress tyrosine hydroxylase, TH), but it is unknown if dopamine specifically in AVPV/PeN kisspeptin cells regulates GnRH neurons. Using Cre-Lox technology, we determined that the selective knock-out of *TH* in kisspeptin cells surprisingly has no effect on puberty, reproductive hormones, or fertility. Thus, despite being highly coexpressed, dopamine in kisspeptin cells is not required for reproduction. Dopamine in these cells likely serves other yet-to-be-identified roles, perhaps unrelated to reproductive neuroendocrinology.

## Introduction

The neuropeptide kisspeptin, encoded by the *Kiss1* gene, stimulates GnRH release and, thus, is necessary for reproduction (de Roux et al., 2003; Seminara et al., 2003; Gottsch et al., 2004; Lapatto et al., 2007; d'Anglemont de Tassigny et al., 2008; Topaloglu et al., 2012). There are two primary hypothalamic populations of *Kiss1* neurons: in the anteroventral periventricular—rostral periventricular continuum (AVPV/PeN) and arcuate (ARC) nuclei (Gottsch et al., 2004; Clarkson and Herbison, 2006). *Kiss1* neurons in the ARC are likely involved in pulsatile gonadotropin secretion and steroid hormone negative feedback, as gonadectomy (GDX) increases ARC *Kiss1* levels, while testosterone (T) or estradiol (E_2_) treatment suppresses ARC *Kiss1* expression (Smith et al., 2005a, b; Kauffman et al., 2007). In contrast to the ARC, in the AVPV/PeN, GDX decreases *Kiss1* expression, while E_2_ treatment increases *Kiss1* expression (Smith et al., 2005a, b; Kauffman et al., 2007). Thus, *Kiss1* in the AVPV/PeN is thought to be involved in mediating E_2_-positive feedback, which triggers the preovulatory LH surge. Supporting this hypothesis, *Kiss1* expression in greater in females than males (males do not show an LH surge), *Kiss1* neurons in the AVPV/PeN express ERα and show increased neuronal activation exclusively during the LH surge, and *Kiss1* KO and *Kiss1*r KO mice cannot exhibit an LH surge even with exogenous E_2_ treatment (Smith et al., 2005a, 2006; Kauffman et al., 2007; Clarkson et al., 2008; Robertson et al., 2009; Dror et al., 2013).

In addition to the sexually dimorphic population of *Kiss1* neurons, the AVPV/PeN also contains a sexually dimorphic population of tyrosine hydroxylase (TH) neurons, with females having more TH cells than males (Simerly et al., 1985). TH is the rate-limiting enzyme in catecholamine synthesis, and AVPV/PeN TH neurons are known to be dopaminergic (Simerly et al., 1985; Scott et al., 2015). These dopaminergic AVPV neurons have both direct and indirect projections to GnRH neurons, indicating AVPV-derived dopamine may influence reproduction (Clarkson and Herbison, 2011; Kumar et al., 2015). However, there is conflicting evidence as to whether dopamine stimulates or inhibits GnRH release. Application of dopamine to medial basal hypothalamic explants increased GnRH release (Rotsztejn et al., 1977; Jarjour et al., 1986). However, intracerebroventricular injections of dopamine antagonists increased the amount of GnRH and LH release during a PMSG-induced GnRH surge, which suggests that dopamine inhibits GnRH release (Sarkar and Fink, 1981). Supporting an inhibitory role of dopamine on GnRH, electrophysiology experiments demonstrate that dopamine can inhibit the firing of GnRH directly, via D1 and D2 receptors (Liu and Herbison, 2013).

Interestingly, in mice, the vast majority of AVPV/PeN *Kiss1* neurons are dopaminergic and coexpress *TH* (Semaan et al., 2010), whereas TH is not localized with kisspeptin in the ARC region (Szawka et al., 2010). However, the function of dopamine produced in AVPV/PeN *Kiss1* neurons is currently unknown. As much as 75% of AVPV/PeN kisspeptin neurons that coexpress TH send fiber projections that appose GnRH neurons (Clarkson and Herbison, 2011; Kumar et al., 2015), which suggests that dopamine signaling arising from AVPV/PeN kisspeptin neurons may directly modulate GnRH secretion, and hence, the neuroendocrine reproductive axis. The current study was designed to assess the effects of selectively deleting *TH* specifically from *Kiss1* cells on puberty onset, hormone levels, and reproduction. Because kisspeptin neurons in the ARC do not coexpress TH, we hypothesized that any effect of removing *TH* from *Kiss1* cells on puberty onset and/or reproduction is attributed to AVPV/PeN *Kiss1* neurons, due to the high degree of colocalization of *TH* and *Kiss1* in the AVPV/PeN. Because dopamine is inhibitory to GnRH neurons, we predicted that puberty onset may be accelerated and/or fertility or fecundity would be enhanced when dopamine is removed from *Kiss1* cells. Though females express more *TH* and *Kiss1* in the AVPV/PeN than males, males still express notable numbers of both celltypes in this region and little is known about the role, if any, of AVPV/PeN *Kiss1* or TH in male puberty or reproduction. Therefore, we studied both sexes to identify any possible sex differences in the reproductive function of *TH* in *Kiss1* cells. To this end, we hypothesized that any observed effects would be sex specific, occurring more in females.

## Materials and Methods

### Animals

**Figure 1.**
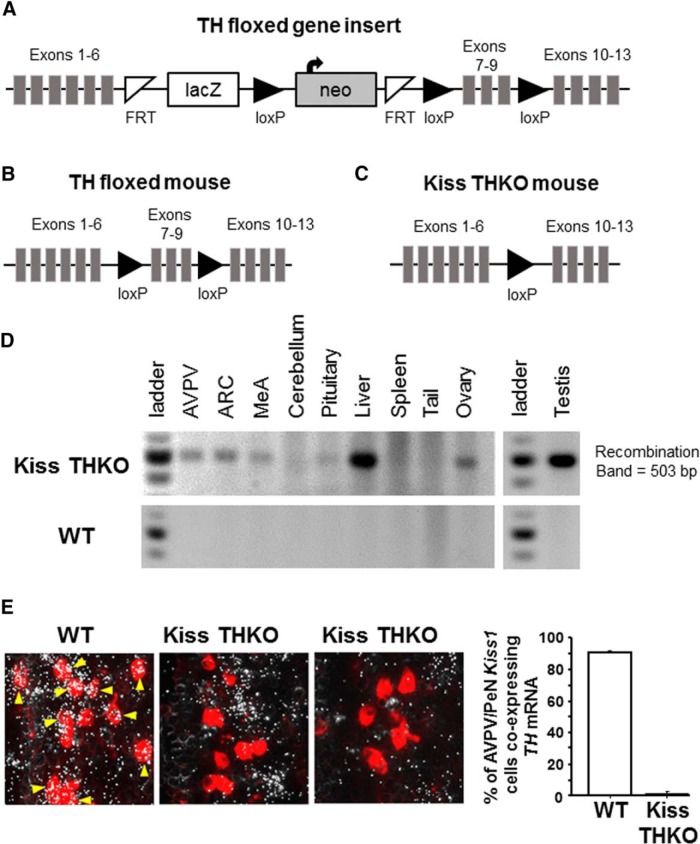
Strategy for knocking *TH* exclusively out of *Kiss1* cells. ***A***, The map of the TH “floxed” gene insert in embryonic stem cells (KOMP repository, UC Davis). Presence of the neomycin cassette in PCR analysis was indicative of germline transmission of the TH flox insert. ***B***, Breeding neomycin positive females to Flp recombinase males removed the lacZ and neomycin cassettes and produced TH flox heterozygous mice. These TH fl/wt mice were then bred to each other to produce TH fl/fl mice. ***C***, KissCre mice were bred to TH fl/fl mice to produce KissCre+ TH fl/fl mice (Kiss THKOs), which lack exons 7-9 of the *TH* gene and thus, a functional *TH* transcript, but only in *Kiss1* cells. ***D***, Gel image of PCR analysis showing Cre-mediated recombination of *TH*, indicated by the 503 bp band representing successful recombination of the *TH* gene, only in tissues known to express *Kiss1* (AVPV/PeN, ARC, MeA, liver, gonad) and not in other tissues (cerebellum, spleen, tail), which are known to not normally express *Kiss1* in Kiss THKO (top) and WT (bottom) mice. ***E***, left, Photomicrograph of *TH* (silver grains) and *Kiss1* (red fluorescence) double label ISH in the AVPV/PeN region of adult female mice. *TH* mRNA is clearly highly expressed in almost all AVPV/PeN *Kiss1* neurons (examples indicated by yellow arrows) of WT mice but absent in virtually all *Kiss1* cells in Kiss THKO mice. Right, Quantification of the percentage of AVPV/PeN *Kiss1* cells coexpressing *TH* in WT and KissTHKO adult female mice (*n* = 4-5/genotype).

We created TH “flox” mice that have loxP sites flanking exons 7-9 of the mouse *TH* gene, a region shared by all transcript variants and which, when ablated, disrupts >50% of the protein-coding sequence of the TH enzyme. Embryonic stem cells with such loxP sites (Fig. 1*A*) were obtained from the KOMP repository at UC Davis (Targeting project #CSD47610; Allele: Th^tm1a(KOMP)Wtsi^). The stem cells were from a C57BL/6N-A^tm1Brd^ mouse strain with an Agouti (A/a) coat color. The local university Transgenic Mouse Core injected the TH flox stem cells into blastocyst stage C57BL6 albino embryos. The blastocysts were then implanted into pseudopregnant females, resulting in chimeric pups. Agouti pups were indicative of germline transmission of the TH floxed gene insert. Tail DNA and PCR analysis of the presence of the neomycin cassette confirmed germline transmission of the TH flox insert. Neomycin-positive females were then bred to Flp recombinase males (purchased from The Jackson Laboratory) to remove the lacZ and neomycin cassettes located between the FRT sites (Fig. 1*A*). Pups of this cross were then genotyped for neomycin, Flp recombinase, and TH flox. Males and females that were neomycin negative/Flp recombinase positive/TH heterozygous were then bred to nonsiblings as adults. From this cross, Flp recombinase negative/TH heterozygous male and female non-siblings were then bred to each other to produce TH fl/fl mice (Fig. 1*B*). TH fl/fl mice were determined to be viable, completely fertile, and had normal body size and weights.

Using Cre-Lox technology, we produced mice lacking *TH* selectively from *Kiss1* cells by mating Kiss1 Cre mice (provided by Dr. Carol Elias) with our TH flox mice. The heterozygous offspring of this first generation were then backcrossed to TH flox mice, resulting in KissCre+ TH fl/fl mice (termed Kiss THKO; *TH* specifically knocked out of *Kiss1* cells) and KissCre- TH fl/fl mice (termed WT or controls; *TH* still present in all cells; Fig. 1*C*). PCR analysis of tail DNA was used to genotype mice (forward primer: CTGCTGATGGTTGGGTTGG; reverse primer: GCGACATCTCTGAATGACC; WT = 182 bp; TH fl = 400 bp) and to test for germline recombination (forward primer: GGAGGAAATTGCTACCTGG; reverse primer: CCCTGCAACATACACTTCACC; WT = 1257 bp; TH fl = 1555 bp; recombination = 503 bp) using the following PCR conditions [95 × 5', (95 × 30”, 53 × 30”, 72 × 30”), repeat × 30 times, 72 × 10']. Any mouse with germline recombination was excluded from the experiments. At weaning (three weeks old), mice were housed two to four per cage on a 12/12 h light/dark cycle and given access to food and water *ad libitum*. All of the experiments were approved by the local Institutional Animal Care and Use Committee.

### Confirmation of conditional TH deletion

To verify Cre-mediated deletion of *TH* in *Kiss1* cells, micropunches of the AVPV/PeN, ARC, and amygdala, as well as small samples of other peripheral tissues, were collected for DNA isolation. PCR analysis was used to confirm that recombination of the *TH* gene occurred in just tissues known to express *Kiss1*, such as the AVPV/PeN, ARC, amygdala, liver, and gonad. In addition, in a separate cohort of adult female ovariectomized (OVX), E_2_-treated (proestrous levels) control and Kiss THKO mice, we used double-label *in situ* hybridization (ISH; see below) to quantify the degree of *TH* and *Kiss1* coexpression in the AVPV/PeN (where *Kiss1* and *TH* normally are highly coexpressed). This confirmed that Kiss THKOs lacked *TH* in AVPV *Kiss1* neurons, unlike controls who still had a high level of coexpression. To ensure the loss of *TH* was specific to *Kiss1* cells, we also counted and compared the number of non-*Kiss1 TH* cells in the AVPV/PeN between WT and Kiss THKO mice (50-60% of all TH cells in the AVPV/PeN do not coexpress kisspeptin and are a distinct population separate from the *Kiss1* cells in this region; Semaan et al., 2010).

### Puberty onset and fertility measures

Beginning at postnatal day (PND) 21, male and female Kiss THKO and WT littermates (*n* = 6-9/sex/genotype) were checked daily for preputial separation, a pubertal marker in males, or vaginal opening (VO), a midstage pubertal marker in females. Following VO, females were checked daily for first estrus (FE, a sign of first ovulatory event, a later pubertal event) based on vaginal smears.

To assess fertility, at eight weeks of age, male and female Kiss THKO mice and WT littermates (*n* = 5-8/sex/genotype) were set up with age-matched C57BL6 breeder partners for eight weeks. During those eight weeks, breeding pairs were examined daily to check for new litters, and the number of pups in each litter was counted on the day of birth. After eight weeks, the breeding pairs were separated and the breeder females were monitored for any new litters for an additional three weeks (i.e., a total of 11 weeks since the initial breeder pairing). We then calculated the percentage of mice having litters, the latency to first litter, the total number of litters, the total number of pups, and the mean number of pups/litter.

### Basal gonadotropin measurements and the E_2_-induced LH surge

To assess basal gonadotropin levels, a blood sample was collected at eight weeks of age from gonad-intact males and females (diestrus) of both genotypes (*n* = 13-15/genotype/sex). Blood serum was sent to the Ligand Assay Core at the University of Virginia to measure LH and FSH (both sexes), as well as testosterone (T) levels (males). LH and FSH levels were quantified using a mouse multiplex assay with a detection limit of 0.24 ng/ml for LH and 2.4 ng/ml for FSH. T levels were measured using an ELISA assay with a lower detection limit of 10 ng/dL.

In a separate cohort of eight-week-old adult female mice, we used a well-established estrogen-positive feedback paradigm (Stephens et al., 2015; Luo et al., 2016) to examine the E_2_-induction of the circadian-timed LH surge. Briefly, females were OVX and given E_2_ SILASTIC implants, which produce constant proestrus-like levels of E_2_ (Dror et al., 2013). Females were then sacrificed two days later, either in the morning at 10 A.M. (*n* = 4-5/genotype) or in the evening at 5:45 P.M., just before lights off (*n* = 9-11/genotype). In mice, the E_2_-induced LH surge occurs only in the evening, around the time of lights off (Robertson et al., 2009). Brains were collected, immediately frozen on dry ice, and stored at -80°C before being sectioned on a cryostat in 20-μm sections. Brain sections were mounted on Superfrost-plus slides in five alternating sets and stored at -80°C until being used for ISH. Blood was also collected at sacrifice and blood serum assayed for LH using a sensitive LH RIA (lower detection limit of 0.04 ng/ml) at the Ligand Assay Core at the University of Virginia.

### Longitudinal body weights and gonad tissue collections

Body weights in gonad-intact mice were measured every three weeks, from 3 to 24 weeks of age, to ensure there were no genotype differences in body weight that may affect fertility (*n* = 4-6/sex/genotype).

In a separate cohort of adult WT and Kiss THKO mice (*n* = 4-8/sex/genotype), gonads were collected at eight weeks of age. Gonads were immediately weighed; the ovaries were then fixed in 4% paraformaldehyde, embedded in paraffin, and cut in 10-µm sections on a microtome. Ovary slices were then mounted onto slides and stained with hematoxylin and eosin to permit the counting of the number of corpora lutea (a marker of ovulation) for each ovary (*n* = 6-7/genotype).

### Single- and double-label ISH

Using an established radio-labeled (^33^P) antisense mouse *Kiss1* riboprobe (0.04 pmol/ml), *Kiss1* levels in the AVPV/PeN were assayed in females (eight weeks old) via single-label ISH, using one of the five sets of brain sections, as previously described (Kim et al., 2013; Di Giorgio et al., 2014; Stephens et al., 2015, 2016). In separate sets of brain sections, double-label ISH analysis was also completed to (1) quantify the degree of *TH* and *Kiss1* coexpression in the AVPV/PeN; and (2) analyze neuronal activation in *Kiss1* and *GnRH* cells during the LH surge, using a *cfos* mRNA induction as an indicator of neuronal activation. Double-label ISH analysis was performed as previously described by combining a radio-labeled (^33^P) *TH* or *cfos* probe (0.04 pmol/ml) and digoxygenin-labeled (DIG) *Kiss1* or *GnRH* riboprobe (1:500) before application to each slide (Dror et al., 2013; Semaan and Kauffman, 2015; Stephens et al., 2015). Microscopy analysis of ISH slides was completed by an observer “blind” to treatment group through the use of an automated silver grains imaging processing system (Dr. Don Clifton, University of Washington). The computer program counts the number of cells, represented as silver grain clusters, as well as the number of silver grains in each cell, representing a semi-quantitative measure of mRNA content per cell (Chowen et al., 1990). For double-label ISH analysis, DIG cells (*Kiss1* or *GnRH*) were identified and outlined using fluorescence microscopy and the grain counting software quantified the number of silver grains (*cfos* or *TH* mRNA) within those cells. Signal-to-background ratios were then calculated for each cell, and cells were considered to be double-labeled if the signal-to-background ratio was > 4.

### Statistical analysis

All data are expressed as the mean ± SEM for each group. Unpaired *t*-tests were used for all puberty onset, basal gonadotropin, gonadal weight, and fertility analyses. Body weight was analyzed using a repeated-measures ANOVA. LH levels during the A.M. and P.M. on the day of the LH surge and quantitative ISH measures were analyzed using a two-way ANOVA and Bonferroni *post hoc* tests. Statistical significance was reached if *p* < 0.05.

## Results

### Verification of conditional deletion of *TH* **from** *Kiss1* **cells**

Using Cre/lox technology, we selectively removed *TH* only from *Kiss1* cells. Confirming proper excision of the *TH* gene in *Kiss1* cells, Cre-mediated recombination of the *TH* gene was detected in several tissues from Kiss THKO mice, specifically in the ARC, AVPV/PeN, MeA, liver, and gonads (all tissues known to normally express *Kiss1*; Fig. 1*D*). Tissues known to not express *Kiss1*, such as cerebellum, tail, and spleen, did not show recombination of *TH*, indicating that recombination was specific to *Kiss1*-expressing cells. To further confirm the conditional knock-out and quantify the degree of *TH* ablation from *Kiss1* cells, we performed double-label ISH for *TH* and *Kiss1* coexpression in the AVPV/PeN brain region. As expected, WT females showed a very high degree of *TH*/*Kiss1* colocalization in the AVPV/PeN, with nearly 90% of their *Kiss1* neurons coexpressing *TH* mRNA. In contrast, Kiss THKO females showed virtually no colocalization of *TH* in *Kiss1* cells in the AVPV/PeN (<3%; *p* < 0.05 vs controls; Fig. 1*E*). To ensure the loss of TH was specific to just kisspeptin cells, we also counted and compared the number of non-*Kiss1 TH* cells in the AVPV/PeN (since approximately half of all the *TH* cells in this region are a separate cell population from the *Kiss1* cells). We found no difference between WT and Kiss THKO mice in the number of non-*Kiss1 TH* cells in the AVPV/PeN (231 ± 20 and 249 ± 13, respectively, *p* = 0.49), indicating the loss of *TH* was specific to *Kiss1* cells.

### Puberty onset and body weights

**Figure 2.**
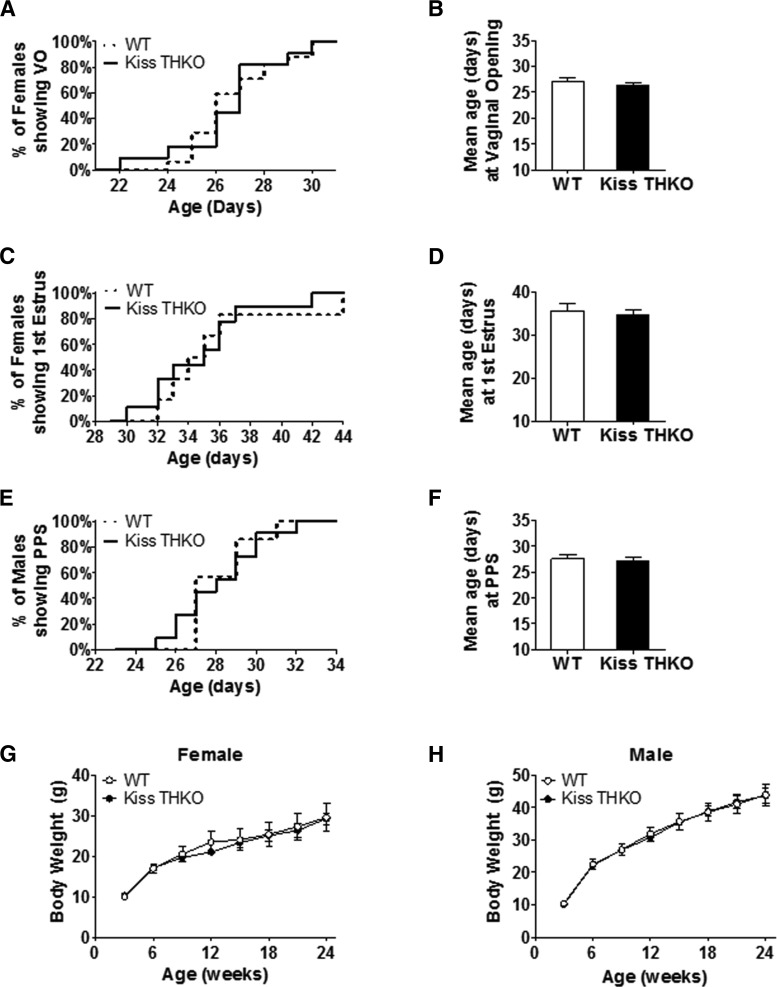
Removal of *TH* selectively from just *Kiss1* neurons does not alter the timing of puberty onset. ***A***, Percentage of females experiencing VO from PND 22 to PND 31. ***B***, Mean age at VO in females. ***C***, Percentage of females experiencing FE from PND 30 to PND 44. ***D***, Mean age at FE in females. ***E***, Percentage of males experiencing preputial separation (PPS) from PND 22 to PND 34. ***F***, Mean age at PPS in males (*n* = 6-9/genotype). ***G***, ***H***, Body weight throughout development and adulthood did not differ between WT and Kiss THKO females (***G***) and males (***H***; *n* = 4-6/genotype).

Because dopamine can inhibit GnRH neurons and puberty onset is governed by GnRH release, we first examined if puberty onset in Kiss THKO mice was altered. For females, neither the timing of VO (midstage pubertal marker) nor FE (late pubertal marker) differed between WT and Kiss THKO mice (Fig. 2*A*,*B* and *C*,*D*, respectively). In males, the pubertal marker of preputial separation was similarly not altered in Kiss THKOs and occurred at a comparable age to controls (Fig. 2*E*,*F*). Body weights did not differ between WT and Kiss THKO mice of either sex throughout puberty (data not shown), nor did BWs differ throughout development and adulthood (Fig. 2*G*,*H*).

### Reproductive hormones and gonad weights

**Figure 3.**
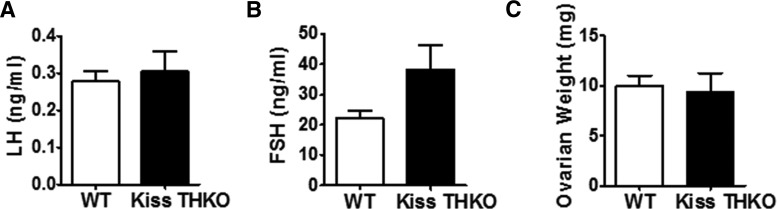
Basal gonadotropin levels and ovarian weights in females did not differ. LH (***A***) and FSH (***B***; *n* = 13/genotype) as well as ovarian weights (***C***; *n* = 6-8/genotype) did not differ between eight-week-old WT and Kiss THKO females.

**Figure 4.**
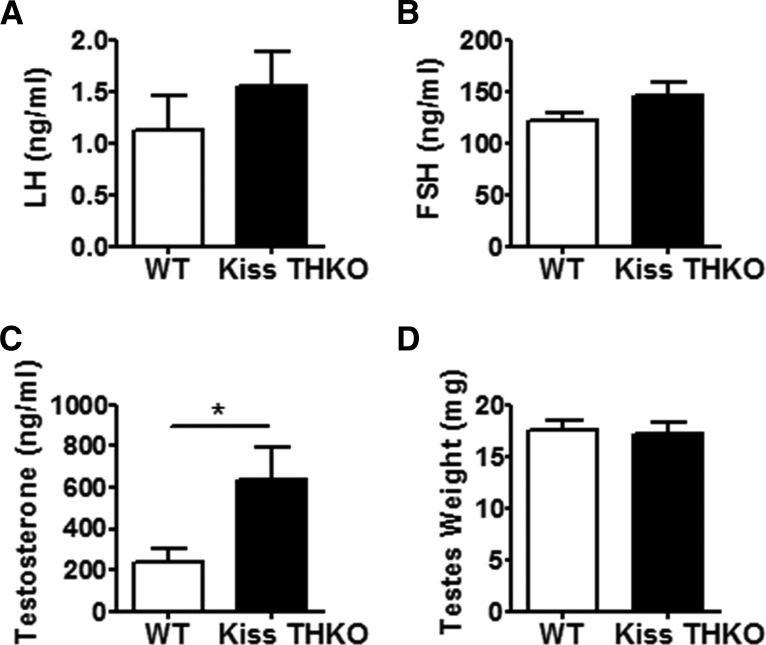
Basal hormone levels and testes weights in eight-week-old WT and Kiss THKO male mice. LH (***A***) and FSH (***B***) did not differ between WT and Kiss THKO males (*n* = 13-15/genotype). T (***C***) levels were significantly higher in Kiss THKO males (*p < 0.05; *n* = 13-15/genotype). However, testes weights (***D***) did not significantly differ between WT and Kiss THKO males (*n* = 6-8/genotype).

Basal LH and FSH levels were comparable between adult WT and Kiss THKO females (all measures from diestrus stage), although there was a nonsignificant trend (*p* = 0.08) for higher FSH levels in Kiss THKO females (Fig. 3*A*,*B*). Ovarian weights also did not differ between WT and Kiss THKO females (Fig. 3*C*,*D*). Kiss THKO males showed a similar outcome as Kiss THKO females, with no significant difference in basal LH levels in comparison to WT littermates males, but a nonsignificant trend for higher basal FSH levels (Fig. 4*A*,*B*). Despite no differences in circulating LH and FSH, Kiss THKO males did have significantly higher circulating serum T levels (Fig. 4*C*; *p* = 0.03); however, testes weight was normal and did not differ between WT and Kiss THKO males (Fig. 4*D*).

### LH surge and ovulation

**Figure 5.**
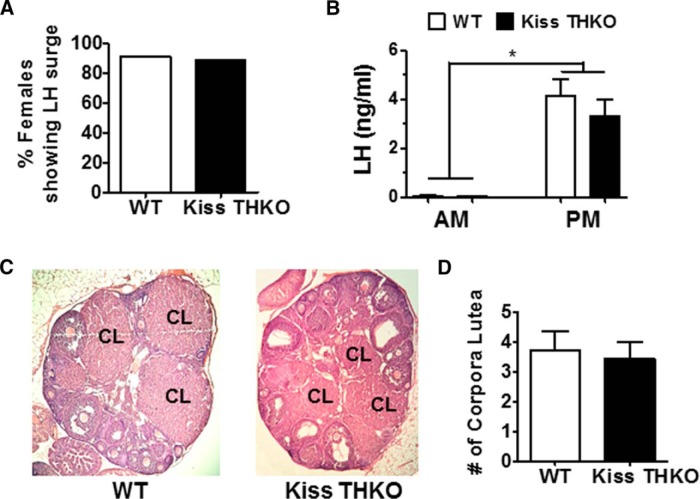
The E_2_-mediated LH surge was not altered in Kiss THKO females. ***A***, Percentage of eight-week-old females having an E_2_-mediated LH surge did not differ between WT and Kiss THKOs (*n* = 9-11/genotype). ***B***, Neither genotype experienced an LH surge in the morning (*n* = 4-5/genotype), but both WT and Kiss THKOs had similar LH levels during the PM LH surge (*n* = 9-11/genotype). ***C***, ***D***, The number of corpora lutea did not differ between genotypes at eight weeks of age (*n* = 6/genotype). *, significant main effect of time, *p* < 0.05.

*TH* is highly coexpressed with AVPV/PeN *Kiss1* neurons, a neural population that mediates E_2_-positive feedback in females to generate the GnRH/LH surge, thereby triggering ovulation. We hypothesized that dopamine produced in these kisspeptin cells may therefore influence the LH surge and ovulation. Using a well-established daily E_2_-induced LH surge paradigm, we found that a similar percentage of OVX + E_2_ WT and Kiss THKO females generated an LH surge (Fig. 5*A*). Both genotypes had very low LH levels in the morning, as expected due to the circadian nature of the LH surge, but had similarly elevated LH levels in the evening, indicative of LH surges (Fig. 5*B*). The number of corpora lutea in the ovaries, indicative of ovulatory events, did not differ between WT and Kiss THKO mice (Fig. 5*C*,*D*).

**Figure 6.**
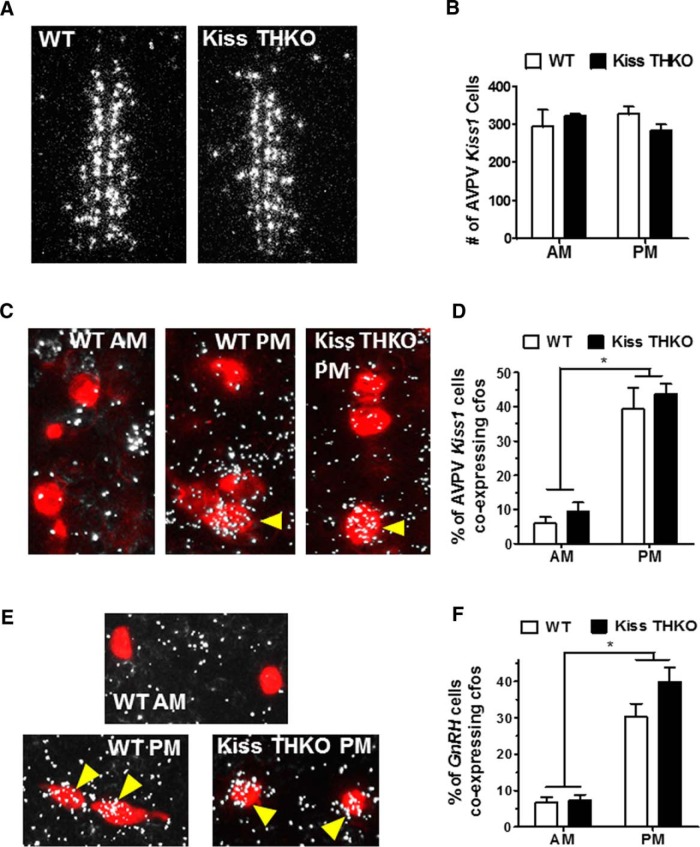
*Kiss1* as well as *cfos* expression in *Kiss1* and *GnRH* cells at the time of the E_2_-mediated LH surge did not differ between WT and Kiss THKO females. ***A***, Photomicrograph of *Kiss1* (silver grains) expression in the AVPV of adult WT and Kiss THKO female mice. ***B***, *Kiss1* levels in the AVPV/PeN of females did not differ between WT and Kiss THKOs. ***C***, ISH photomicrograph of *cfos* (silver grains), a measure of gene activation, and *Kiss1* (red fluorescence) colocalization (indicated by yellow arrows) in the AVPV/PeN of adult female mice in the PM (at the time of the LH surge). ***D***, *cfos* expression in *Kiss1* cells was low in the A.M. and elevated in the P.M. in both WT and Kiss THKOs. ***E***, Photomicrograph of *cfos* (silver grains) and *GnRH* (red fluorescence) coexpression (indicated by yellow arrows) in the OVLT of adult female WT and Kiss THKO mice. ***F***, *cfos* expression in *GnRH* cells was low in the A.M. and elevated in the P.M. (at the time of the LH surge) in both WT and Kiss THKOs. *, significant main effect of time, *p* < 0.05.

We performed single-label ISH for *Kiss1* in the AVPV/PeN to ensure that removal of *TH* from *Kiss1* cells did not alter AVPV/PeN *Kiss1* expression. The number of AVPV/PeN *Kiss1* cells in WT and Kiss THKO mice did not significantly differ (Fig. 6*A*,*B*). Neuronal activation, measured by *cfos* coexpression, of *Kiss1* cells in the AVPV/PeN was significantly greater during the LH surge (P.M.) in comparison to the A.M. (*p* < 0.001) and did not differ between WT and Kiss THKO mice at either time point (Fig. 6*C*,*D*). Similarly, GnRH neuronal activation, measured by *cfos* induction in *GnRH* cells, was significantly greater in the evening than in the morning for both WT and Kiss THKO females (*p* < 0.001), with a similar magnitude of *cfos* coexpression for the two genotypes (Fig. 6*E*,*F*).

### Fertility and fecundity

**Figure 7.**
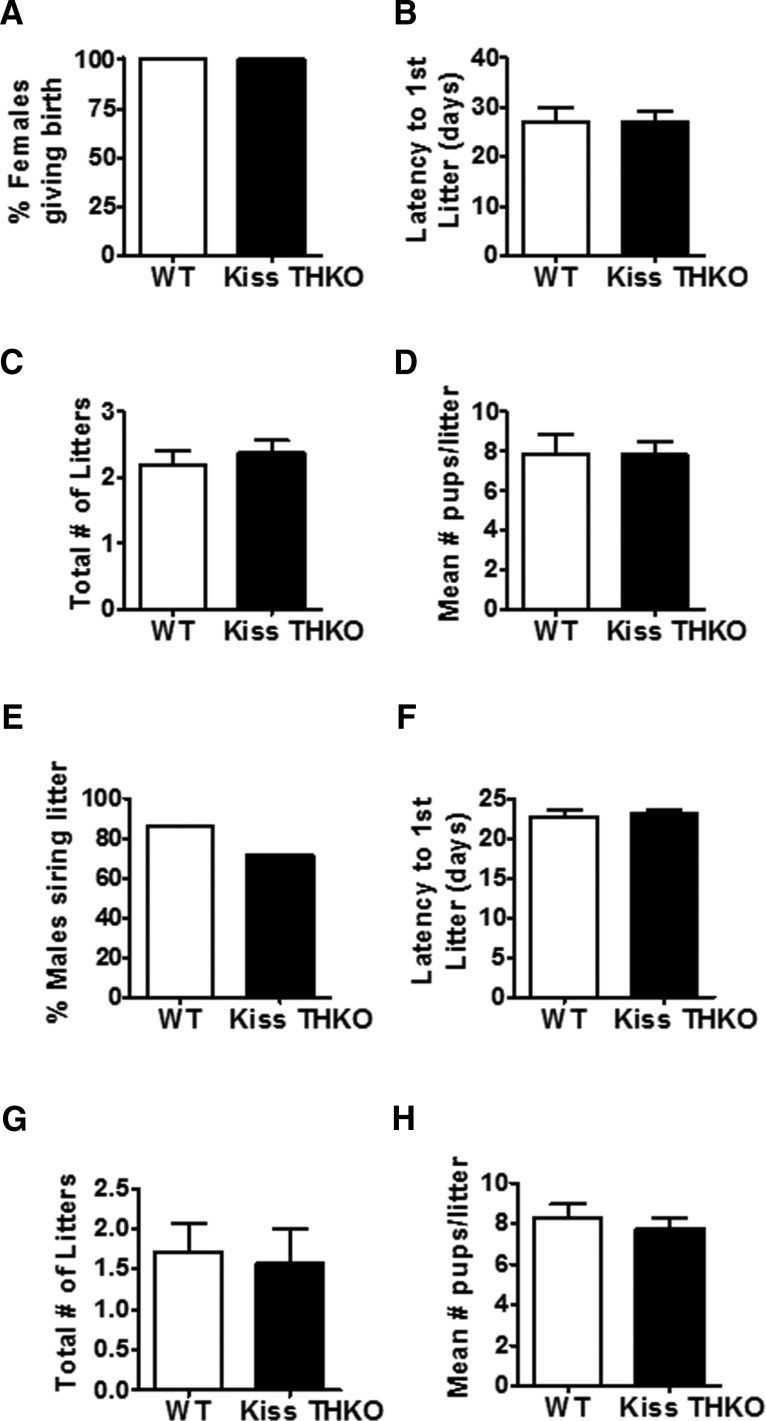
*TH* in *Kiss1* cells is not required for normal fertility in females (***A--D***) or males (***E--H***). ***A***, Percentage of WT and Kiss THKO females that had a litter during the 11-week fertility assessment. ***B***, Mean latency (days) to first litter after pairing with the male. ***C***, Mean total number of litters during the 11-week fertility study. ***D***, Mean number of pups/litter (*n* = 5-8/genotype). ***E***, Percentage of males siring at least one litter during the 11-week fertility assessment (*n* = 7/genotype). ***F***, Mean latency (days) between the initial pairing with the female and when the first litter was born; measure only includes males that sired a litter. Mean total number of litters each male sired (***G***) and the mean number of pups/litter (***H***).

Because of dopamine’s inhibitory actions on GnRH, we hypothesized that removal of *TH* from *Kiss1* cells would lower inhibition on GnRH neurons and thereby enhance specific parameters of fertility and/or fecundity (e.g., time to producing litters, total numbers of litters generated, number of pups per litter). We found that Kiss THKO mice of both sexes were completely fertile. In females, there were no significant genotype differences in the percentage of females giving birth, the latency to first litter, the total number of litters, and the mean number of pups/litter between WT and Kiss THKO mice (Fig. 7*A-D*), indicating fertility and fecundity were not enhanced in Kiss THKO females. Males showed a similar outcome, with no alterations in any fertility or fecundity measures in Kiss THKO mice (Fig. 7*E-H*).

## Discussion

Kisspeptin regulates puberty and reproduction by directly stimulating the release of GnRH. Kisspeptin neurons in the AVPV/PeN are sexually dimorphic and participate in E_2_-mediated positive feedback and the preovulatory LH surge. The vast majority of kisspeptin cells in the AVPV/PeN express *TH* mRNA and protein (Semaan et al., 2010; Clarkson and Herbison, 2011), which suggests that dopamine, like kisspeptin, in the AVPV/PeN may be important for governing GnRH secretion and perhaps participating in E_2_-positive feedback. To test this possibility, we used Cre-lox technology to selectively ablate *TH* from *Kiss1* cells. Surprisingly, despite a practically complete knock-out of *TH* from *Kiss1* cells, we found that all aspects of puberty, reproductive hormone secretion, and fertility were normal in Kiss THKOs. This suggests that dopamine synthesized in *Kiss1* cells is not required for normal puberty and reproduction.

Because (1) dopamine inhibits GnRH neurons (Liu and Herbison, 2013), (2) *TH* and *Kiss1* are highly colocalized in the AVPV/PeN (Semaan et al., 2010), and (3) kisspeptin neurons in the AVPV/PeN regulate E_2_-positive feedback (Smith et al., 2006; Robertson et al., 2009), we hypothesized that puberty completion may be accelerated and fertility/fecundity may be enhanced, primarily in females since males have a much smaller *Kiss1/TH* population (Kauffman et al., 2007; Semaan et al., 2010). Surprisingly, despite a virtually complete ablation of *TH* levels in *Kiss1* cells, puberty measures were comparable between WT and Kiss THKO mice in both sexes. Puberty onset is initiated by an increase in pulsatile GnRH and LH release, and most studies have implicated ARC kisspeptin neurons, rather than AVPV/PeN kisspeptin neurons, in the regulation of pulsatile GnRH release. Thus, it would stand to reason that altering AVPV/PeN but not ARC kisspeptin neurons would not dramatically impact puberty onset. However, puberty completion in females is dependent on achieving first ovulation (measured by FE), and this would be dependent on proper AVPV/PeN kisspeptin neuron function underlying the LH surge process. Despite this, we found that FE in females lacking dopamine in AVPV/PeN kisspeptin neurons occurred at a normal age and was not hastened.

As with puberty, we were surprised to find that fertility and fecundity measures were completely normal in Kiss THKO mice of both sexes. We found no differences in basal circulating gonadotropin levels, multiple fertility measures, or the induction and magnitude of an LH surge between adult WT and Kiss THKO females. This indicates that *TH* (i.e., dopamine) in AVPV/PeN *Kiss1* cells is not required for male or female reproduction and proper regulation of the reproductive neuroendocrine axis. Our findings support recent data showing that ablation of a majority (75%) of all AVPV TH cells, not just *TH* in *Kiss1* cells, had no effect on estrous cycles or female reproductive success (male fertility not studied; Scott et al., 2015). However, the percentage of AVPV kisspeptin cells that remained in that study was not determined, and it is highly possible that enough kisspeptin cells (perhaps also 25% like the TH cells) remained to sustain female reproduction.

Because AVPV/PeN *TH* and *Kiss1* expression levels are markedly lower in males, and male reproduction is likely regulated primarily by ARC kisspeptin neurons (which do not coexpress TH; Szawka et al., 2010), we hypothesized that puberty onset and fertility would be similar in WT and Kiss THKO males. Indeed, as in females, puberty onset, basal gonadotropin, and fertility were all normal in Kiss THKO males. However, despite comparable LH and FSH levels and normal testes weights, circulating T levels were significantly higher in Kiss THKO males. Since this increase in T was not accompanied by higher gonadotropin levels, we speculate that this represents an effect occurring specifically within the testes. The testes express both kisspeptin and TH (Frungieri et al., 2000; Davidoff et al., 2005; Anjum et al., 2012), and as such, TH/dopamine may be significantly ablated within testicular cells (though it is unknown if kisspeptin and TH are in the same testis cell types). It is possible that such a decrease in local dopamine may alter testicular T synthesis, although this remains to be tested in future studies. Regardless, there was no significant difference in any fertility measure between WT and Kiss THKO males, suggesting that the elevated T did not enhance reproduction in the latter group.

Most AVPV/PeN *Kiss1* cells coexpress *TH* (Semaan et al., 2010), yet our present data indicate that *TH*, and hence, dopamine, in *Kiss1* cells is not required for proper regulation of puberty and fertility in females and males. Thus, the function of dopamine signaling arising from *Kiss1* cells currently remains unknown. Recent data suggests that some AVPV TH neurons project to the PVN and activate oxytocin neurons, thereby increasing oxytocin secretion (Seymour et al., 2017). This AVPV TH neuron to PVN oxytocin circuit may play a role in maternal behavior and/or lactation (Scott et al., 2015; Seymour et al., 2017). Thus, it is possible that dopamine in AVPV/PeN *Kiss1* cells is involved in oxytocin release or plays a role in regulating maternal behavior. However, our Kiss THKO females successfully carried full pregnancies, had normal parturition times, and normal litter sizes, suggestion oxytocin was likely unaffected. Future research is needed to directly evaluate this hypothesis and to further examine additional functions of dopamine coexpressed in *Kiss1* cells.

## Synthesis

Reviewing Editor: Jeffrey Blaustein, University of Massachusetts Decisions are customarily a result of the Reviewing Editor and the peer reviewers coming together and discussing their recommendations until a consensus is reached. When revisions are invited, a fact-based synthesis statement explaining their decision and outlining what is needed to prepare a revision will be listed below. The following reviewer(s) agreed to reveal their identity: Victor Navarro, Michael Lehman

There was consensus that, even though the study reports expected, primarily negative results, it provides a significant advance in the field. There was also consensus that the following issues needed attention.

1. Was the verification of TH ablation from AVPV Kiss1 neurons done after supplementation with E2? There is no mention about E2 replacement in the results. This could be a potential limitation because not all Kiss1 neurons would be labeled.

2. Is TH in the AVPV regulated by sex steroids? This needs to be taken into consideration when looking at co-expression for the validation of the model.

4. Please, specify the post hoc test performed in every statistical analysis.

5. Figure 1D needs to indicate what the Kiss THKO band corresponds to.

6. Readers would appreciate a rationale for the inclusion of males in this study.

7. It would strengthen the verification of the selectivity of the KO if the authors were also to provide some data (images and/or numbers) showing that TH expression was unaffected in cells not expressing kisspeptin (e.g., non-Kiss1 TH-positive neurons in the AVPV or A12 dopamine cells in the ARC). Of course, this would be more of a concern if effects on reproductive function had been observed, but nonetheless, would be good for completeness of the study. It would also help address the possibility that compensatory increased TH expression in other populations that are afferent to GnRH neurons (see below) might have contributed to the lack of effect on reproduction.

8. The recent Nature paper (Scott et al., 2015) cited by the authors (lines 267-268), reporting that ablation of all AVPV TH cells had no effects on estrous cycles would seem to be at odds with strong evidence that AVPV kisspeptin cells play a key role in the regulation of the preovulatory GnRH/LH surge. Even with 10% of kisspeptin cells remaining (those not expressing TH), one would think that ablation of 90% of AVPV Kiss1 neurons would have a significant effect on the timing and/or amplitude of the surge, and consequent estrous cyclicity and reproductive success. How do the authors reconcile that, is there evidence for that much redundancy in the circuitry?

9. The authors speculate that because dopamine may have inhibitory actions on GnRH cells, removal of TH from Kiss1 cells "may enhance fertility and/or fecundity" (line 230). Please explain how this could be assessed, since animals were already fertile.
